# Advancing the Care Experience for Patients Receiving Palliative Care as They Transition From Hospital to Home (ACEPATH): Phase 2 of Codesigning an Intervention to Improve Hospital‐to‐Home Transitions for Patients and Family Caregivers

**DOI:** 10.1111/hex.70331

**Published:** 2025-07-11

**Authors:** Madeline McCoy, Taylor Shorting, Vinay Kumar Mysore, Edward Fitzgibbon, Jill Rice, Meghan Savigny, Natalie C. Ernecoff, Marianne Weiss, Shirley H. Bush, Daniel Vincent, Meaghen Hagarty, Geneviève Lalumière, Rex Pattison, Mona Kornberg, Maya Stern, Kerry Kuluski, Colleen Webber, Adrianna Bruni, Tara Connolly, Sarina R. Isenberg

**Affiliations:** ^1^ Bruyère Health Research Institute Ottawa Canada; ^2^ Consultant New York New York USA; ^3^ The Ottawa Hospital Research Institute Ottawa Canada; ^4^ The Ottawa Hospital Ottawa Canada; ^5^ Department of Medicine, Division of Palliative Care, The Ottawa Hospital University of Ottawa Ottawa Canada; ^6^ Bruyère Health Ottawa Canada; ^7^ Consultant Vancouver Canada; ^8^ RAND Corporation Pittsburgh Pennsylvania USA; ^9^ Marquette University Milwaukee Wisconsin USA; ^10^ Regional Palliative Consultation Team (RPCT) Ottawa Canada; ^11^ Caregiver Partner Ottawa Ontario Canada; ^12^ Patient Partner Ottawa Ontario Canada; ^13^ Institute for Better Health Trillium Health Partners Mississauga Canada; ^14^ University of Toronto, Institute of Health Policy, Management and Evaluation Toronto Canada; ^15^ Carleton University, Accessibility Institute Ottawa Canada

**Keywords:** care transitions, codesign, health services research, palliative care, patient and caregiver engagement

## Abstract

**Background:**

Although many people nearing the end of life wish to die at home, many patients experience re‐hospitalisation and hospital death. No end‐of‐life hospital‐to‐home interventions have been developed with patients and caregivers, and none have been tested in Canada. Through an iterative, participatory design approach, we codesigned an intervention in partnership with potential users of the final intervention: patients, family caregivers (FCs) and healthcare providers (HCPs).

**Objective:**

This study (ACEPATH) aimed to use a patient, FC and HCP engaged codesign process to continue to iterate and refine an intervention for transition from hospital to home in preparation for a pilot implementation.

**Methods:**

The codesign process consisted of: (1) Development of codesign workshop (CDW) materials; (2) CDWs with patients and/or their FCs, who iterated our team's previously developed checklists and reference materials; (3) Low‐fidelity prototyping sessions with hospital and community HCPs, who provided feedback on the low‐fidelity prototype, the guidebook (that combined the refined checklists and guides) and identified HCPs to facilitate the guidebook; and (4) High‐fidelity prototyping sessions entailed simulated interactions between an HCP and a patient/FC using the intervention, accompanied by discussion for feedback.

**Results:**

Participants identified several areas for refinement to enhance the relevance, clarity and acceptability of the guidebook intervention. Patients and FCs refined and organised questions into specific ‘moments’ that would be helpful for conversations with HCPs during their transition home. HCPs identified social workers, hospital home care coordinators and community home care coordinators as the best fit for facilitating completion of the guidebook at three moments (preparing to leave the hospital, immediately before discharge and getting comfortable at home).

**Conclusions:**

We successfully codesigned a guidebook for hospital‐to‐home transitions that was amenable to patients, FCs and HCPs. The next steps will entail piloting the guidebook to evaluate its acceptability, appropriateness, feasibility, costs and fidelity.

**Patient or Public Contribution:**

Patients and FCs who had lived/living experiences with hospital‐to‐home transitions near the end of life participated in codesign workshops and high‐fidelity prototyping sessions. We used codesign to ensure the final intervention was aligned with participants' needs and experiences and would hopefully improve aspects of the hospital‐to‐home transition that are important to them.

## 1 Introduction

### 1.1 Hospital‐to‐Home Transitions and Shortcomings of Existing Interventions

Many people nearing the end of life wish to die at home [1, 2, 3, 4] and receive care at home instead of in a hospital. However, only about half of Canadians die at home or in the community [1, 2, 3, 4]. Additionally, many patients experience emergency department visits, re‐hospitalisation or hospital death following hospital discharge [1, 2, 5, 6, 7]. Previous studies describe gaps in transition coordination, information gaps between hospital and community healthcare providers (HCPs), and a need for improved systems for communication between HCPs [8, 9, 10, 11, 12, 13]. Most patients and family caregivers (FCs)^1^ feel unprepared for the complex, disjointed and challenging hospital‐to‐home transition [8, 12].

Most hospital‐to‐home interventions have been resource‐intensive, involving care coordinators and nurse navigators [10, 14, 15, 16, 17]. These interventions often focus on hospital readmission as the primary outcome, which is not a person‐centred measure [17]. Furthermore, existing interventions have generally focused on acute care hospitals rather than subacute care facilities [18]. To date, no end‐of‐life interventions in Canadian acute or subacute care settings have been developed in partnership with patients and FCs. For a more detailed overview of the literature in this regard, see our previous paper [11].

### 1.2 Participatory Design: CDWs, Low‐Fidelity Prototyping Sessions and High‐Fidelity Prototyping Sessions

Codesigned interventions are more likely to meet the needs of patients and their FCs [19, 20, 21], as they enable patients and FCs to share their perspectives [22] while fostering collaboration, empowerment and ownership during intervention development [21, 23, 24]. A recent systematic review explored the use of codesign for developing palliative care interventions, highlighting the need for standardisation of the codesign process [25]. In adherence with the codesign best practice checklist, this study furthers the use of codesign in palliative care. Further delineated in our ‘Methods’ section, patients, CGs and HCPs were engaged in all stages of intervention and outcome measures development [25]. Our person‐centred and iterative approach incorporated feedback from potential end users (patients, FCs and HCPs) to create effective interventions [25, 26]. We detail our process for collecting feedback to re‐design and refine the guidebook intervention [25].

CDW participants confirmed the content of the intervention design. Low‐fidelity and high‐fidelity prototyping sessions developed and tested intervention concepts at ‘various levels of resolution’ or fidelity [26], ranging from ideation and feedback on early conceptualisations of the intervention (i.e., low fidelity) [26] to a simulated interaction between participants to gauge the use of a mockup of the intervention in a real‐world setting (i.e., high fidelity) [26].

## 2 Objective

To use a patient‐, FC‐ and HCP‐engaged codesign process to iterate and refine an intervention for transition from hospital to home in preparation for a pilot implementation at two sites.

This paper is part of a broader study (Advancing the Care Experience for patients receiving Palliative care as they Transition from hospital to Home [ACEPATH]) on hospital‐to‐home transitions^2^ for patients receiving a palliative approach to care in acute and subacute settings,^3^ [8, 10, 17, 27, 28] wherein we are developing and testing an intervention [11]. Previously, at an acute care site (Site 1), we codesigned components of a preliminary intervention [11]. Our current paper delineates refinement of the intervention design using codesign at a subacute care site (Site 2), as well as low‐fidelity prototyping sessions and high‐fidelity prototyping sessions (Sites 1 and 2) [26].

## 3 Methods

### 3.1 Study Design

**Figure 1:**
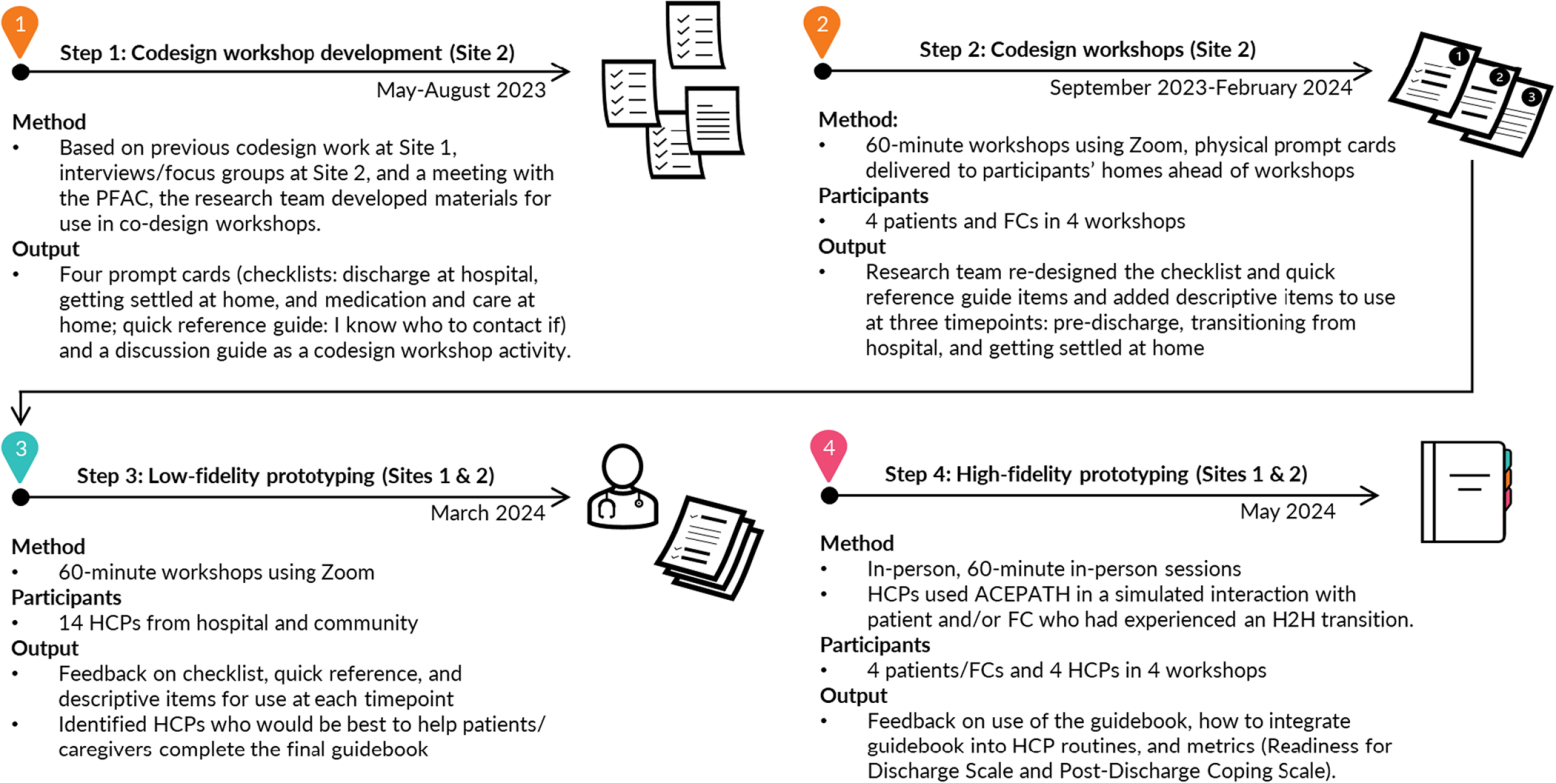
Codesign process steps—development of codesign workshops, codesign workshops, low‐fidelity prototyping sessions and high‐fidelity prototyping sessions. Site 1 = acute care hospital, Site 2 = subacute inpatient facility, PFAC = Patient and Family Advisory Council, FC = family caregiver, HCP = healthcare provider, H2H = hospital to home.

**Figure 2:**
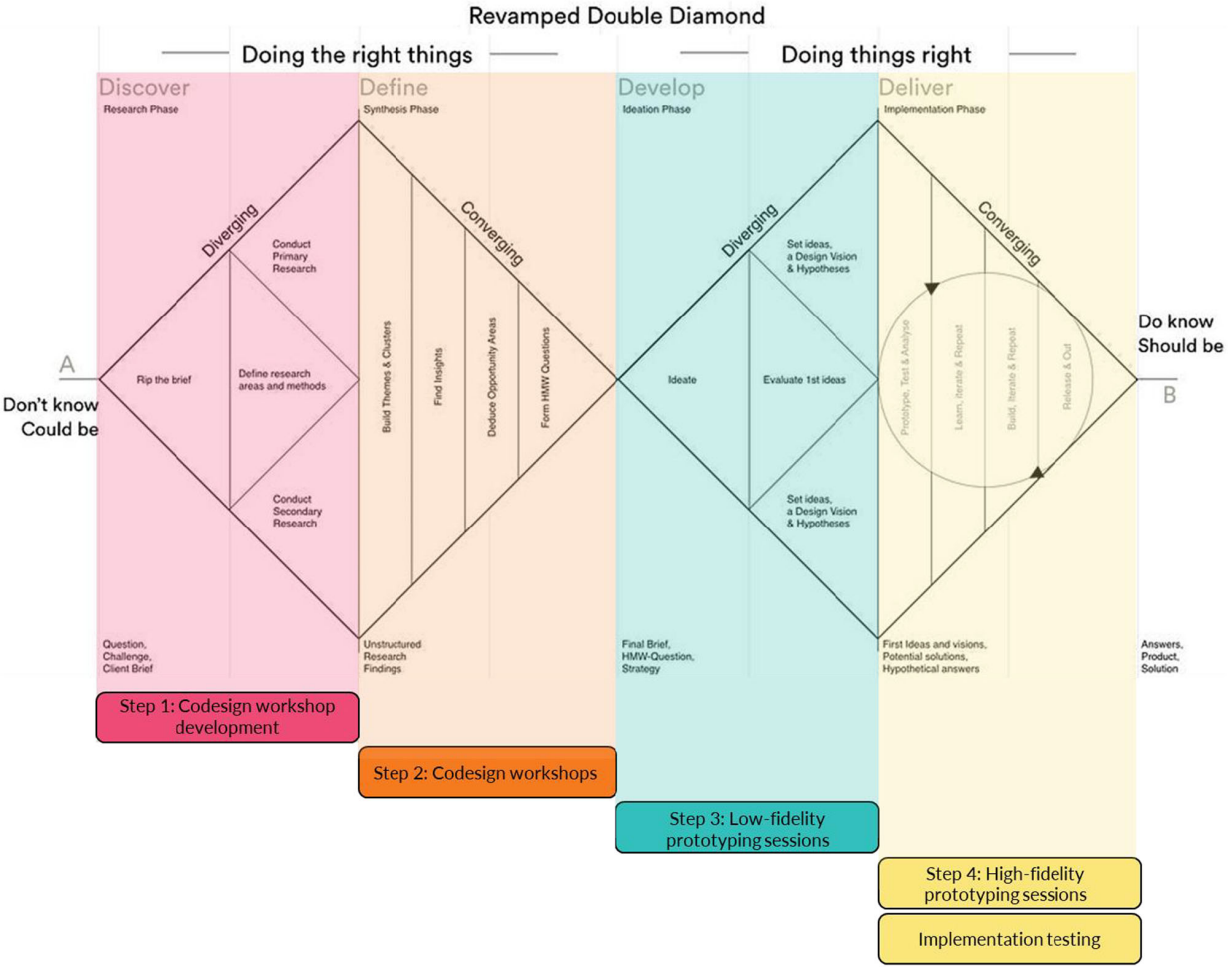
Project steps mapped onto the Double Diamond Framework [29, 30, 31].

During previous CDWs (Site 1), patients and FCs expressed a preference for simple, targeted documents to support their transition from hospital to home. HCPs in previous low‐fidelity prototyping sessions (Sites 1 and 2) provided feedback on four prototypes. Our previous paper details our process for developing early intervention concepts [11]. The current paper focuses on our next steps in collecting feedback and re‐designing our prototype intervention that will be tested using a pilot study. We followed the codesign best practice checklist and Double Diamond Framework [25, 29, 30, 31]; Figures 1 and 2 illustrate the four project steps and their alignment with the Double Diamond Framework.

Ottawa Health Science Network Research Ethics Board (OHSN‐REB) at Site 1 granted exemption as this study met their criteria for a quality improvement project. Bruyère Health Research Ethics Board at Site 2 approved the study (REB number: M16‐23‐033). The research team consisted of health services researchers, palliative care physicians and nurses, a design researcher (V.K.M.), a communications designer (M.Sa.) and research support staff. The Patient and Family Advisory Council (PFAC) was involved throughout the project.

### 3.2 Setting

Site 1, The Ottawa Hospital, located in Ottawa, Ontario, Canada, is a tertiary academic acute care hospital with a 4‐bed acute palliative care unit and an interprofessional palliative care consult team. Site 2, Bruyère Health, located in Ottawa, Ontario, Canada, is a subacute facility with a 31‐bed palliative care unit and a physician‐only palliative care consult team.

### 3.3 Step 1: Development of CDWs (Site 2)

#### 3.3.1 Approach to CDW Development

Our previous work at Site 1 led to feedback on preliminary interventions [11] and broader suggestions surrounding intervention needs from interviews and focus groups at Site 2 (manuscript under review). In Step 1, we developed CDWs, aligning with the Discover—Research Phase of the Double Diamond Framework [29, 30, 31]. We had several working meetings to synthesise findings into prompt cards. Using Miro [32], the team collaborated to visualise themes with virtual sticky notes and text boxes [33, 34]. We used affinity mapping [34] to organise potential checklist items and quick reference guide items into themes for the prompt cards.

We finalised the prompt cards and CDW discussion guide based on the PFAC's feedback. M.Sa. designed the cards considering accessibility.

### 3.4 Step 2: CDWs (Site 2)

#### 3.4.1 Recruitment

HCPs from the patient's circle of care identified patients and/or FCs based on the inclusion criteria:

1. Patients who had experienced a hospital‐to‐home transition while receiving a palliative approach to care at the study site or patients who planned to transition home to receive comfort‐focused care and/or their FCs.
2. Patients and FCs able to communicate in English or French and are 18+ years.
3. Participants must have the capacity to consent.

Additional recruitment strategies included: posters, handouts, an Intranet post, presenting at a hospital joint‐discharge meeting, circulating a recruitment email to clinical staff, and emailing weekly reminders to palliative care physicians and a social worker. Previous interview and focus group participants (manuscript under review) were also invited to participate.

To include a diverse representation of patients/FCs in the CDWs, we recruited from seniors' support services, seniors' programmes and FC support networks in Ottawa serving equity‐deserving groups (e.g., those without English or French as a first language or with low income). We contacted 16 organisations, and interested organisations shared our recruitment letter through their networks.

#### 3.4.2 Data Collection

We scheduled CDWs following discharge. Participants completed a demographics questionnaire via Microsoft Forms before the CDW. We conducted 60‐min, virtual CDWs using MS Teams. Patients and FCs could participate independently or in a patient–FC dyad. We recorded the CDWs and took notes.

#### 3.4.3 Approach to CDWs

In Step 2, our CDWs aligned with the Define—Synthesis Phase of the Double Diamond Framework [29, 30, 31, 34] and engaged patients and FCs in conversation about the checklist and quick reference guide content. We delivered physical prompt cards to participants' home addresses for participants to review beforehand (Figure 3).

Figure 3Prompt cards and discussion guide for codesign workshops (Site 2). Introduction. Situate the participant in the larger project we're working on, and centring the value of their lived expertise. Framing this as a ‘redesign’ of the existing checklist concept as a narrative way of making space for codesigned contributions. Used a focused narrative approach at this moment of the current codesign process as opposed to having them go through all the work to date. Re‐imagination prompt is the focus of the conversation to make this process more co‐creative. Prompt Card 1. Included questions about the activity itself (e.g., ‘would you even want a checklist?’) to make space for participants to depart from the prompt in ways that make the most sense to them. Varied items in the list at different levels of technicality and varied what the item is checking off (e.g., achievements like ‘I met with’ or prompts like ‘I have a plan for’) to see what items are more useful for participants. Prompt Card 2. Repeating the general structure to build familiarity. This list is built like more of a preparation checklist; participants are invited to compare and contrast the lists to discuss what's important for them in the design of the intervention. Prompt Card 3. Focus on comparative inquiry while maintaining the same general structure of the interview so far. Beginning to ask questions around the form and design of the final output (e.g., ‘would you want to have the checklist be on sheets of paper like this?’). Prompt Card 4. Continue to increase questions on form and interactive elements (i.e., asking how HCPs could answer these questions and when) as participants are more comfortable with the pattern of going through each sheet and evaluating the list itself. Note. 911 = emergency phone number used in Canada.

**Figure:**
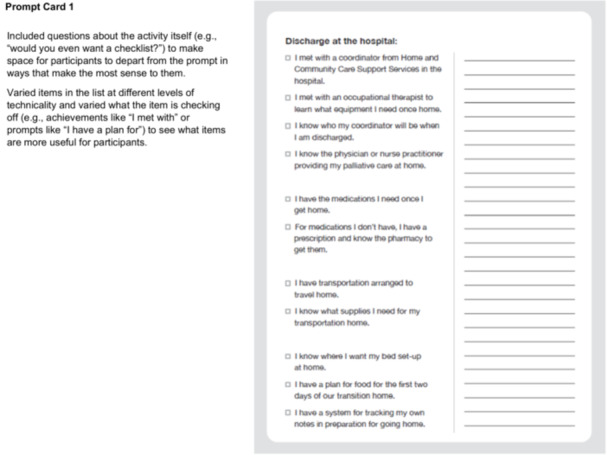

**Figure:**
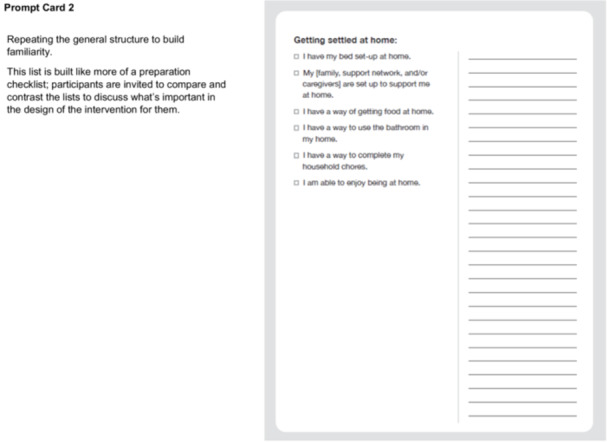

**Figure:**
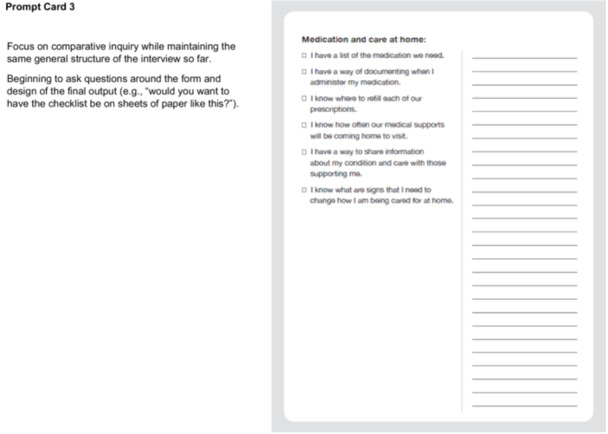

**Figure:**
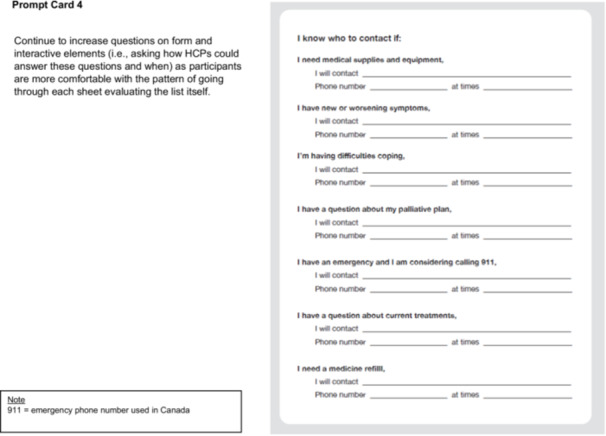

VKM facilitated the CDWs, following the CDW discussion guide (Figure 3).

We asked participants to re‐imagine their hospital‐to‐home transition experience and consider:

1. Content: what could be added, changed or removed from the three checklists (corresponding to each key moment in the hospital‐to‐home transition) and quick reference guide? Helpful information to receive during the transition without being overwhelming.
2. Design: were checklists/quick reference guide a fitting intervention? Was a paper intervention preferred? To promote ideation, we informed participants that the intervention could be something different if it did not fit their needs.
3. Timing: when would items from the checklists/quick reference guide be useful to review with an HCP throughout the hospital‐to‐home transition?
4. Facilitator selection: which type of HCP could be fitting for facilitating conversations with patients/FCs using the intervention?

#### 3.4.4 Analysis of CDWs

We reviewed CDW notes and, using Miro [32] and affinity mapping, we visualised themes [33, 34]. We organised content changes into items that could be added, changed or removed. As a group, we discussed CDW participants' suggestions on the best timing for reviewing individual checklist/quick reference items and different HCPs' roles in facilitating the intervention and answering questions in‐hospital and the community. V.K.M. and M.Sa. created a low‐fidelity prototype of the guidebook, combining checklist and quick reference guide items. This prototype served as an early concept for how components could be integrated into a single guidebook, designed to help HCPs provide important information and contacts to patients/FCs during key moments in the hospital‐to‐home transition [26, 35].

### 3.5 Step 3: Low‐Fidelity Prototyping Sessions (Sites 1 and 2)

#### 3.5.1 Recruitment

We invited co‐investigators, home care coordinators and participants from our previous study to a low‐fidelity prototyping session. Co‐investigators forwarded details to colleagues who coordinated discharge or cared for patients during the hospital‐to‐home transition.

#### 3.5.2 Data Collection

We conducted virtual low‐fidelity prototyping sessions using MS Teams. We recorded the sessions and took notes. Employees and their direct supervisors were not included in the same sessions to encourage participants to speak openly.

V.K.M. led the sessions, presenting the low‐fidelity prototype (Figure 4) and encouraging feedback on:

1. Modifications to the content of the guidebook.
2. Facilitator role: which type of HCP would be best suited to engage with patients/FCs to review the guidebook, assist in completion of the transition planning content for the three transition moments (preparing to leave the hospital, immediately before discharge and getting comfortable at home), and answer patients'/FCs' questions?
3. Information overload: how could we avoid patients/FCs feeling overwhelmed?

Figure 4Low‐fidelity prototype.

**Figure:**
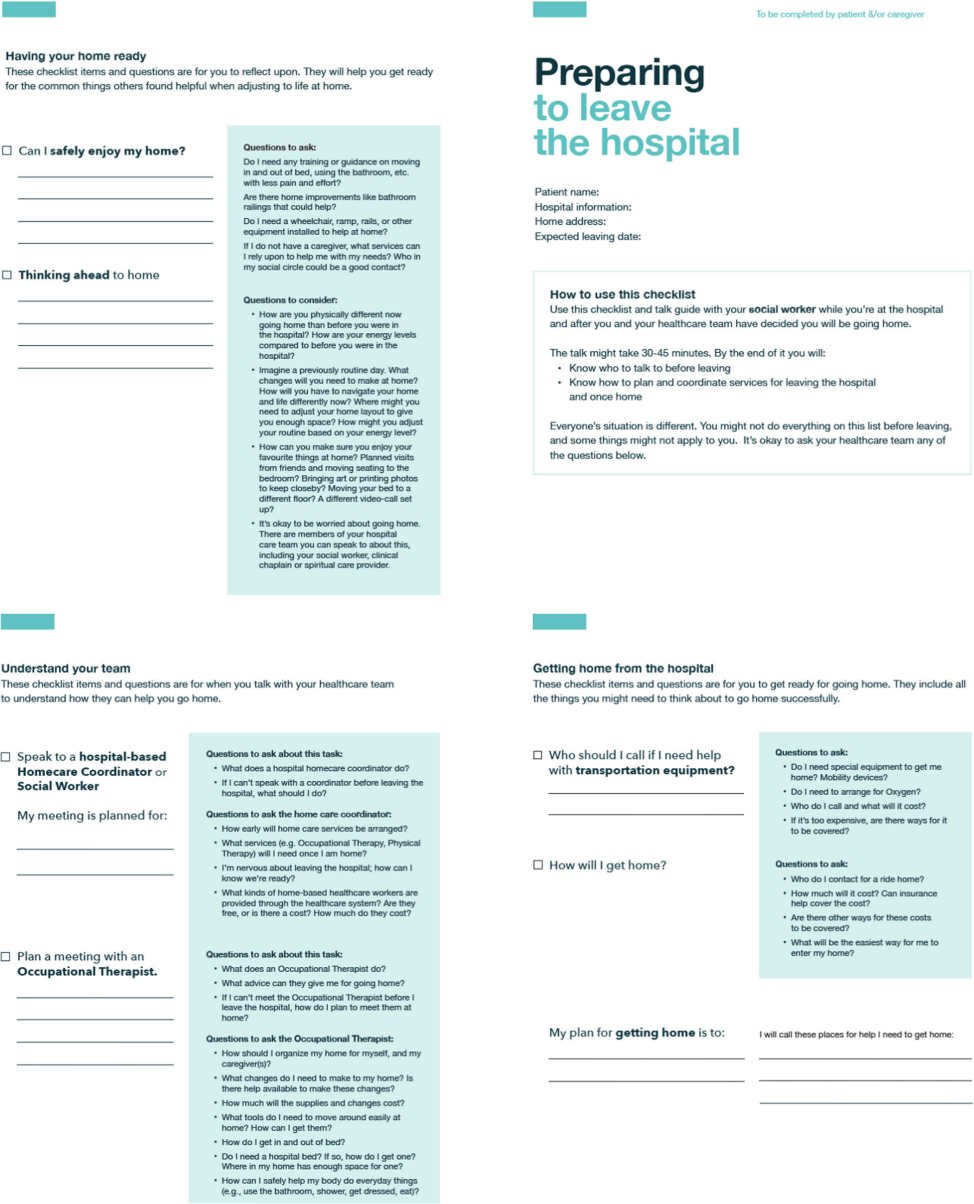

**Figure:**
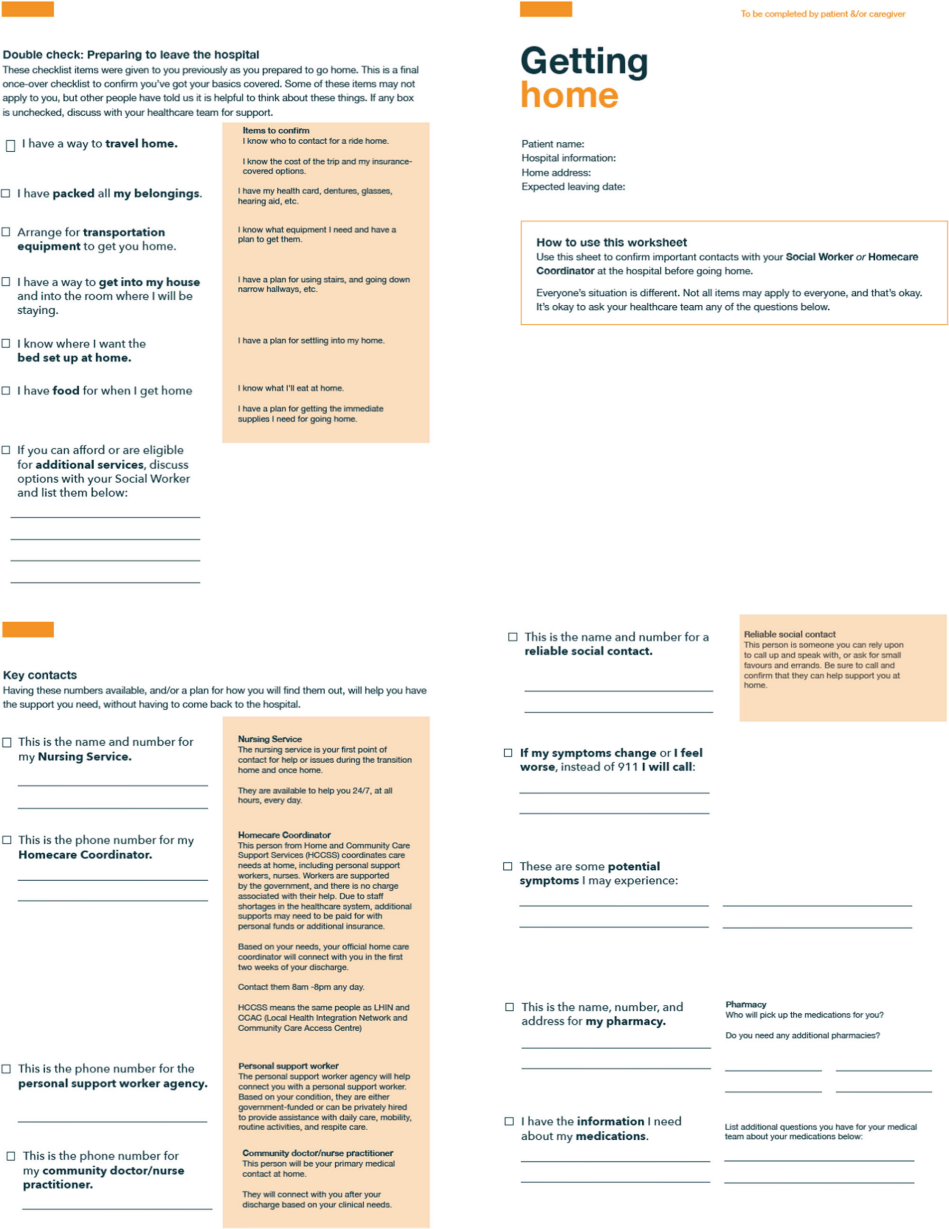

**Figure:**
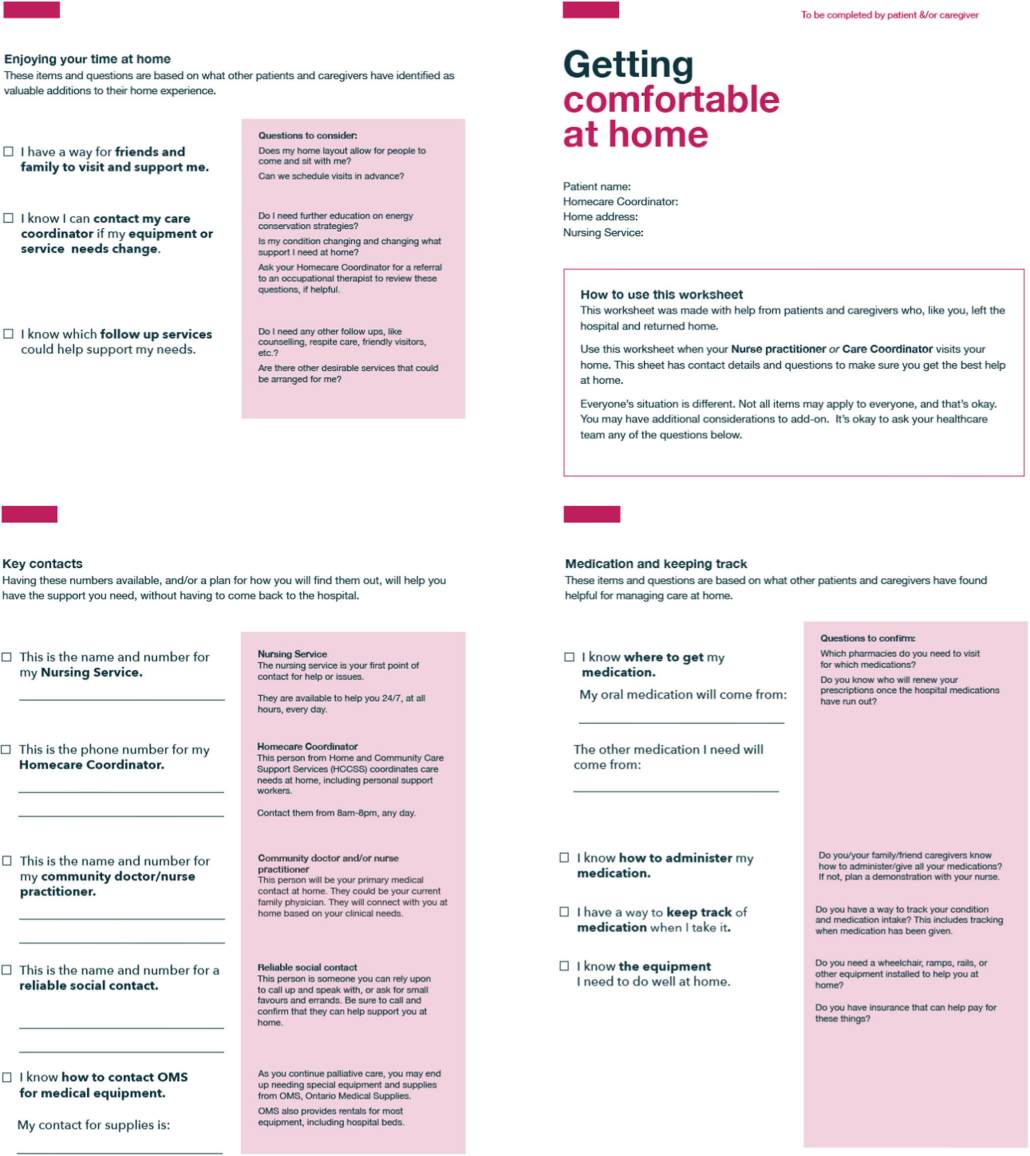

#### 3.5.3 Analysis of Low‐Fidelity Prototype Feedback

In Step 3, our low‐fidelity prototyping sessions aligned with the Develop—Ideation Phase of the Double Diamond Framework [31] and engaged HCPs about the operationalisation of the intervention. After each session, we consolidated feedback and M.Sa. updated the prototype. Using affinity mapping, we organised feedback into themes within the three topics of modifications to the content to include at each time point, discussion of who would be most fitting for the facilitator role at each time point and suggestions for reducing information overload [34]. V.K.M. and M.Sa. made iterative modifications based on working sessions with the rest of the research team. The PFAC and broader team of co‐investigators and collaborators provided feedback at a quarterly team meeting. V.K.M. and M.Sa. developed a high‐fidelity prototype to represent the look, feel and functionality of the final guidebook [26, 35].

### 3.6 Step 4: High‐Fidelity Prototyping Sessions (Sites 1 and 2)

#### 3.6.1 Recruitment

We sent emails to social workers, occupational therapists and home care coordinators at both sites and in the community. Previous patient/FC participants were invited to sessions. We matched a patient and/or FC who had been discharged from Site 1 or 2 with an HCP who worked at the same site, so they participated in the same session.

#### 3.6.2 Data Collection

V.K.M. led in‐person high‐fidelity prototyping sessions. HCPs were encouraged to role‐play a discharge conversation with patients/FCs using the guidebook. Patients/FCs were asked to identify any unclear points or missing questions. HCPs were asked to describe how the intervention could be incorporated into their workflow and identify unanswered questions and who could address these gaps.

#### 3.6.3 Analysis of High‐Fidelity Prototyping Feedback

In Step 4, our high‐fidelity prototyping sessions aligned with the Deliver—Implementation Phase of the Double Diamond Framework [31]. The research team had working sessions to discuss modifications to the guidebook. The team discussed how to limit information overload and incorporate the process for facilitation of the guidebook into HCPs' current routines. V.K.M. and M.Sa. revised the guidebook in preparation for an accessibility assessment, the final step before finalising its content.

The Accessibility Institute at Carleton University in Ottawa, Ontario, Canada, reviewed the guidebook from a disability‐related end‐user perspective. The research team held working sessions to discuss suggested changes to ensure the guidebook could be used by diverse patients and FCs with various accessibility needs.

## 4 Results

### 4.1 Step 1: Development of CDWs (Site 2)

#### 4.1.1 Summary of Findings

Through affinity mapping, the team identified checklist items to guide patients/FCs in conversations with HCPs during three key moments of the hospital‐to‐home transition: preparing to leave the hospital, immediately before discharge and getting comfortable at home. PFAC members emphasised the importance of conversation with HCPs and knowing questions to ask HCPs. PFAC members shared that patients/FCs frequently feel overwhelmed during the hospital‐to‐home transition and need to easily access information.

#### 4.1.2 Synthesis of Findings Into Materials for the Next Phase

The four prompt cards were large and high‐contrast for those with limited vision and printed on cardstock for accessible handling [36, 37].

We organised checklist items into three prompt card categories: (1) items to be checked off before discharge from hospital (prompt card 1—discharge at hospital); (2) items to check off to facilitate getting settled at home (prompt card 2—getting settled at home); and (3) items to check off in relation to medication and care at home (prompt card 3—medication and care at home). Checklist items on prompt cards varied between meetings with HCPs that could be checked off (e.g., ‘I met with___’), plans put in place (e.g., ‘I have a plan for____’), and knowledge about next steps (e.g., ‘I know how I'm getting home’). The quick reference guide contained important contacts and scenarios to contact them (prompt card 4—I know who to contact if___).

V.K.M. and M.Sa. designed the prompt cards and CDW discussion guide to help the team understand information patients/FCs wanted to know at any point during their transition. The CDW discussion guide elicited conversation on how to refine checklist and quick reference guide items based on patient and FC experiences (Figure 3).

### 4.2 Step 2: CDWs (Site 2)

#### 4.2.1 Participant Demographics

Two patients and two FC participants participated in four virtual CDWs. Most participants were 65+ years (*n* = 3; 75%) and identified as women (*n* = 2; 50%). All participants preferred to speak English and lived in an urban area.

#### 4.2.2 Summary of Findings

In Step 1, we had three checklists and one quick reference guide. In Step 2, we reorganised checklist items, items to prompt conversation, questions to ask HCPs and key contacts under the three moments during the hospital‐to‐home transition (preparing to leave the hospital, getting home and getting comfortable at home). Patients/FCs discussed the information they would have wanted to know before the transition. Participants' feedback was categorised into themes:

1. Content
  See Table 1 for an overview of CDW participants' suggested changes. Participants highlighted opportunities for clarifying roles of HCPs in the hospital and community. They also liked having space for notes. Participants emphasised the importance of respecting cultural differences in conversations, such as varying approaches to discussing death and end‐of‐life care.
2. Design
  All participants liked the checklist and quick reference guide intervention and emphasised their preference for a paper intervention (vs. a digital intervention). Participants raised concerns about what would happen if checklist items could not be completed before discharge. For example, was the item not applicable or are they missing information/resources? Patients and FCs wanted to be guided through the intervention with opportunities to ask questions and receive resources, especially regarding the transition process and managing expectations before discharge home. For example, one participant highlighted the importance of clarifying HCP roles in the hospital and the community, especially care coordination.
3. Timing
  Participants emphasised the importance of starting discharge preparation early to ensure home care services were in place at the time of discharge home. They also wanted the intervention in advance of conversations with their healthcare team to review and note questions. Thinking back to their transition experience, participants felt unaware of the questions they should ask HCPs. One participant described the importance of reviewing items when FCs are present, as the patient might not retain all information.
4. Facilitator Selection

**Table 1:** Feedback from CDWs on items to add, modify and remove and other comments.

Checklist: Discharge from the hospital
Items to add
− Is the patient at risk being at home? − Is the patient safe at home/at home alone? − Can the patient feed themselves? − Is long‐term care a better option for this patient? − Is there space for the patient to be at home (i.e., does furniture need to be moved/downsized or do personal items need to be organised to make space)? − Is the patient mobile? − Have you met with a social worker? − How the medications are delivered from the pharmacy and whether they do blister packs
Items to modify
− ‘I know what supplies I need for my transportation home’ (Specify what the word ‘supplies’ entails) − Transportation isn't just a checklist box; sometimes, more conversation is needed − Modify the food item so that it is food for the first week
Items to remove
− Items that raise expectations and are not realistic
Other
− Ensuring that conversations respect cultural differences

Checklist: Getting settled at home
Items to add
− Are you worried about transfers? − Are you worried about cleanliness? − Are you worried about getting in/out of bed? − Are you content? − Are you able to complete transfers to the shower, commode and so forth? − Would a list of resources or a training session be helpful to you? − What are you going to do if your infrastructure at home falls apart? − What supports/services are you eligible for? If you are eligible for 2 h of PSW care per day, what are you going to do for the other 22 h? − Many tasks that suddenly need to be done at once at discharge, making people aware of this somehow − Adding a part about thinking about money, how to organise paperwork for bills and healthcare, and setting up family members' access to accounts, if needed − Would want blank pages after the checklist to take more notes
Items to modify
− Clarify the item ‘I can use the bathroom at home’ à ‘Do I have a way to manage my bodily functions/personal hygiene?’ − Use the bathroom on my own (typo)

Checklist: Medication and care at home
Items to add
− Have you met with the pharmacist/pharmacy team? − I know what medications I need − I know what supports exist to help me ensure my medication regimen (e.g., blister packs) − Do you have a relationship with your pharmacist? − I know what the signs are that I need to change the way I am being cared for (e.g., is this serious enough to call 911/What should I look for in my patient to flag this concern/Should I call a doctor/nurse practitioner?) − I have a way to have pills organised, for example, someone to organise pills or pharmacy blister packs, resources from the pharmacy − I have a way to refill prescriptions

Quick reference guide: I know who to contact if___
Items to add
− Who would you ask about transportation to medical appointments? − What if you need to talk about transitions to nursing homes? Where would you get info, who would you talk to? − Day‐to‐day tasks—even foot care, because you can't bend over
Items to modify
− Clarify what ‘at times’ means − ‘Questions about my palliative plan’ is too general—there are many questions to raise about transportation, nursing homes, care in the home and next steps in care

*Note:* 911 = emergency phone number used in Canada.

Abbreviation: PSW = personal support worker.

Participants identified social workers, nurses, occupational therapists and in‐hospital care coordinators (who coordinate discharge) as key HCPs who could answer many of these questions. In the community, participants felt home care coordinators or nurses would be key HCPs.

#### 4.2.3 Synthesis of Findings Into Materials for the Next Phase

We restructured the three checklists and quick reference guide into one guidebook with three sections for the transition moments. The team discussed the information patients/FCs needed at each moment and what questions/prompts could ensure they receive this information.

Moment 1: Preparing to leave the hospital. Checklist items for hospital meetings and key points to discuss with the healthcare team to prepare for transition home. Checklist items included considerations for getting home successfully and ways to prepare for adjusting to life at home.

Moment 2: Getting home. Items to ‘double‐check’ when discharge is imminent. Items would confirm patients/FCs have all important contact information and ensure all supports are in place before discharge (e.g., do you have all your belongings, is transportation organised, is a bed set up at home and is food available at home?).

Moment 3: Getting comfortable at home. A quick reference guide with space for contact details to get help at home. Additionally, checklist items ensure patients/FCs had a method for medication tracking and suggest ways to enjoy their time at home in a new way.

Patients'/FCs' identified HCPs to help with navigation, which prompted the research team to explore how HCPs would assist in the intervention facilitation.

Based on team discussions, V.K.M. and M.Sa. created a low‐fidelity prototype for Step 3 (Figure 4).

### 4.3 Step 3: Low‐Fidelity Prototyping Sessions (Sites 1 and 2)

#### 4.3.1 Participant Demographics

We held six virtual low‐fidelity prototyping sessions with 14 HCPs (Table 2). Over 50% of participants worked in the community (*n* = 8; 57%) and had training in palliative care (*n* = 12; 86%).

**Table 2:** Participant demographics for low‐fidelity prototyping sessions (Sites 1 and 2).

Low‐fidelity prototyping session participants	
Healthcare providers (*n* = 14)	
Role	Number (Per cent)
Physician	3 (21%)
Nurse	3 (21%)
Nurse Practitioner	0 (0%)
Care Coordinator	2 (14%)
Social Worker	1 (7%)
Physiotherapist	1 (7%)
Occupational Therapist	2 (14%)
Spiritual Care Provider	1 (7%)
Administrative Staff	1 (7%)
Other	0 (0%)
Training in PC	
Yes	12 (86%)
No	2 (14%)
Hospital or Community?	
Hospital	5 (36%)
Community	8 (57%)
Other	1 (7%)
Preferred language of communication	
English	14 (100%)
French	0 (0%)
Location	
Urban (more than 1000 people)	14 (100%)
Rural (less than 1000 people)	0 (0%)
Years in practice	
0–9	3 (21%)
10–19	4 (29%)
20–29	2 (14%)
30–39	3 (21%)
40+	1 (7%)
No response	1 (7%)

Abbreviation: PC = palliative care.

#### 4.3.2 Summary of Findings

1. Modifications to content
  Participants provided feedback to improve the clarity of checklist items, questions for HCPs and quick reference contacts. For example, clarifying that patients should consult their healthcare team about their specific equipment needs and the first contact upon arrival home, and removing redundant checklist items across time points.
  Participants also suggested that the language should be reassuring and indicate that, although transitions are overwhelming, support is always available. Additionally, participants recommended we avoid setting expectations about timelines for meetings with HCPs, for example, removing that home care coordinators in the community would reach out to patients/FCs within 2 weeks after discharge. Coordinators prioritise patients based on the urgency of their need for services (i.e., patients with urgent care needs will be contacted sooner). Accurate expectations would prevent patients/FCs from worrying if they did not receive a phone call within a certain time frame.
2. Facilitator role
  Social workers were identified as key HCPs to assist patients/FCs at the first moment (preparing to leave the hospital), social workers/hospital home care coordinators were identified as the best fit for the second moment (getting home), and community home care coordinators were identified as the best fit for the third moment (getting comfortable at home). However, participants agreed that key HCPs would not be able to answer all guidebook questions and could connect patients/FCs to other HCPs for more specific questions. For example, an occupational therapist could answer questions about preparing their home, and a pharmacist could answer questions about a patient's medication. Participants raised concerns that every patient's situation is different, and some meetings with HCPs do not happen in the hospital.
3. Information overload

Participants noted reducing repetition would help prevent patients/FCs from feeling overwhelmed by perceiving the guidebook as three separate handouts. Additionally, participants suggested the social worker bring the guidebook to patients/FCs when discharge becomes an option. Like patient/FC participants, HCPs recommended that patients/FCs review the guidebook at their own pace before a social worker provides an overview.

#### 4.3.3 Synthesis of Findings Into Materials for the Next Phase

The team identified three goals within the three moments: conversing, patients/FCs received important questions to ask; confirming, essential checklists to complete with the assistance of HCPs; and connecting, a quick reference guide for patients and FCs to understand their healthcare team and contacts at different points in their transition. An overview of the hospital‐to‐home transition process was added, with key items for conversing, confirming and connecting outlined at each moment.

We determined there would be a key HCP who would facilitate different parts of the guidebook that were relevant to their role and direct patients/FCs to HCPs who could answer specific questions or arrange services. This would also give patients/FCs time and space to absorb information and ask questions.

The research team ensured the language throughout the guidebook reassured patients/FCs that they were not alone. We balanced patient/FC CDW feedback with HCP feedback when clarifying checklist items, questions for the healthcare team and quick reference contacts. Additionally, we added a QR code to a list of resources to avoid adding more pages.

V.K.M. and M.Sa. modified the low‐fidelity prototype into a high‐fidelity prototype for Step 4.

### 4.4 Step 4: High‐Fidelity Prototyping Sessions (Sites 1 and 2)

#### 4.4.1 Participant Demographics

We held four in‐person high‐fidelity prototyping sessions (two at Site 1 and two at Site 2) with one patient, three FCs and four HCPs. Three sessions had a total of one patient, three FCs and three HCPs. In one session, the HCP had a research team member act as a patient/FC. Most patients/FCs were 65+ years (*n* = 3, 75%) and identified as women (*n* = 3, 75%). All preferred to speak English and lived in an urban area.

One care coordinator, one occupational therapist and two social workers participated; two were trained in palliative care. All HCP participants were < 65 years, identified as women, preferred to speak English (although some were bilingual) and worked in an urban hospital.

#### 4.4.2 Summary of Findings

##### 4.4.2.1 Patient and FC Feedback

Participants suggested an overview of the HCPs involved in the patient's care in the hospital and community. They also suggested reducing the length of the guidebook and clarifying the goal of each section, as it might be overwhelming alongside the many handouts provided in the hospital. When imagining the interaction between HCP and patient/FC, participants reiterated that they wanted an opportunity to review the guidebook on their own before speaking to an HCP. Finally, FC participants highlighted the need for resources to assist them during this stressful time.

##### 4.4.2.2 HCP Feedback

HCPs discussed how to integrate the guidebook into their workflow, suggesting it be introduced during the initial meeting with the patient/FCs and revisited during follow‐up meetings closer to discharge. Thus, the guidebook could be revisited to clarify questions patients and FCs might have as they meet with more HCPs. Consistent with feedback from low‐fidelity prototyping sessions, HCPs noted that not all patients/FCs meet with every HCP listed, so the guidebook should be adaptable to different situations.

##### 4.4.2.3 Accessibility Assessment

The Accessibility Institute provided a report highlighting areas for improvement, including clearer and more consistent language, better navigation, and improved formatting with more readable fonts and greater contrast between text and background colours. They also suggested training HCPs to support patients and caregivers with diverse needs while using the guidebook.

#### 4.4.3 Synthesis of Findings Into Materials for the Next Phase

High‐fidelity prototyping sessions in Step 4 mapped onto the Double Diamond Framework *Deliver—Implementation Phase* [30].

Takeaways from the high‐fidelity prototyping sessions were to provide more orientation and outline the HCPs involved in each moment. We incorporated an organisational chart and glossary of the hospital and at‐home HCPs. We clarified the goal of each section in the guidebook: the first section introduces the healthcare team and discusses needs and changes that will happen at home; the second reviews what to expect and to double‐check they have everything needed for discharge; and the third focuses on the home healthcare team and settling into life at home.

We refined the guidebook to align with HCPs' workflow and rephrased sections to better reflect the conversation that already happens between HCPs, patients and FCs. We outlined steps if a meeting could not occur with an HCP before discharge.

The research team incorporated supportive language to reassure patients and FCs that this transition might feel overwhelming, but they will be prepared, and support from their healthcare team is available (e.g., contact information and knowing who to call when situations arise at home). Finally, we used inclusive language that involves both the FC and the patient in the discussion or a patient/FC using the guidebook on their own (i.e., we used the general term ‘you’ instead of ‘patient’ or ‘caregiver’).

Based on the accessibility assessment, we referred to our intervention as ‘the guidebook’ and ensured the content was free of medical jargon. We used consistent terms for the three moments to improve navigation. We ensured the font sizes and contrast were improved for people with low vision. Finally, HCPs will be trained to accommodate diverse learners and patients/FCs with different accessibility needs (e.g., supporting the use of an audio‐recorder instead of writing notes or having someone take notes instead of the patient).

The final version of the guidebook can be found here: https://www.isenberglab.com/hospital-to-home-resource-hub.

## 5 Discussion

Codesign is increasingly used in health services research, combining patient‐centred approaches to healthcare delivery, patient and FC empowerment, and design research concepts [24, 25]. Previous research has highlighted the potential for codesign research to align researcher goals and end‐user needs, while integrating interventions into the healthcare system [25, 38].

Our study followed the codesign best practice checklist and the Double Diamond Framework to engage patients, FCs and HCPs in defining the challenges with hospital‐to‐home transitions for those experiencing a palliative approach to care and to develop a solution to the problem [25, 29, 30, 31]. By centring patients and FCs in the intervention design process and considering their lived/living experience, interventions can better meet the needs of patients and FCs [19, 22, 25, 39]. Recently, a team in Toronto developed a plan for codesigning, implementing and evaluating a digital solution to support hospital‐to‐home transitions for older adults with complex care needs [40]. In contrast, our study focused on hospital‐to‐home transitions for patients experiencing a palliative approach to care, and our findings have pointed to patient's and FC's preference for a paper solution. Patients and FC involvement in intervention development ensures the intervention meets the needs of end users [25, 40].

As highlighted by patient/FC participants, breakdown in communication between patients, FCs and HCPs contributes to poor discharge outcomes and overall distress [8]. Other codesigned patient‐oriented initiatives have emphasised the importance of patient‐centred approaches in improving hospital discharge through partnership between patients, FCs and HCPs (e.g., the Patient Oriented Discharge Summary Tool) [40, 41, 42, 43]. Patient/FC participants in our study and PFAC members wanted an intervention that was patient‐ and FC‐facing; our intervention aims to empower patients and FCs to have conversations with HCPs [44]. Participants were actively involved in designing questions, checklist items and quick reference items that they would have found useful during the transition [25].

In contrast, HCPs provided feedback on the operationalisation of the intervention. Previous interventions have been developed by HCPs for use by HCPs [45, 46], which can lead to poor uptake as it is seen as another step added to their workload. Our high‐fidelity prototyping sessions involved role‐playing to imagine how the guidebook could be integrated into HCPs' current routines, aiming for an intervention that met the needs of patients and FCs while being feasible for HCPs [47, 48]. Furthermore, we framed the hospital‐to‐home intervention to improve on the current discharge process, rather than insinuating HCPs were providing inadequate care, a notion cited by clinical guideline implementation strategies [49].

Finally, we have highlighted the value of using codesign to improve healthcare processes and create system‐level change [25, 50, 51]. The success of a health services intervention depends on consideration of scarce resources and overall sustainability of an intervention in the current healthcare system [24, 25, 52]. Thus, our codesigned guidebook works within the constraints of the healthcare system, involving HCPs in the codesign process. This level of HCP involvement can help ensure the intervention is incorporated into the HCP workflow.

Next, we will pilot our intervention and test acceptability, appropriateness, feasibility, costs and fidelity at both sites. Our intervention requires buy‐in from HCPs and administrators to help facilitate the guidebook process. HCP partners, who are paid team members, will facilitate the guidebook during the implementation study. Our goal is to learn how to best integrate the intervention into HCPs' workflow to develop a sustainable process for regular clinical practice beyond our study.

## 6 Strengths and Limitations

Our study has several strengths. We developed an effective codesign process by customising the well‐established Double Diamond Framework [29, 30, 31] to fit the goals of our project. We centred patients and FCs in the process and involved PFAC members throughout.

We held in‐person high‐fidelity prototype sessions in Step 4. We incorporated a physical prototype that participants could use to role‐play. Previous literature highlights in‐person engagement in supporting a more energetic and valuable interaction amongst the patient/FC and HCP participants, compared to virtual formats [47, 48]. Given the finite time and energy level available to participants with life‐limiting diseases [53, 54], all sessions were for 60 min.

We also encountered challenges during the study. Very few home discharges took place at Site 2, so only a small number of patients and FCs were eligible to participate. In‐person sessions could result in missing the perspectives of patients/FCs who could not travel. However, we wanted to emulate the in‐person discharge process closely.

Our research ethics board required a multistage recruitment process (approach, verbal consent to contact, and referral by the patient's circle of care), which meant the research team could not connect with participants before discharge. We contacted patients/FCs post‐transition and re‐engaged participants from earlier phases, but attrition occurred due to death and loss to follow‐up. Many HCPs who would be implementing the intervention were not engaged until high‐fidelity prototyping sessions, limiting their early input. Finally, HCP participants in the high‐fidelity prototyping sessions all worked in hospitals, limiting feedback on the at‐home portion of the guidebook.

## 7 Conclusion

This paper builds on our previous work to provide a detailed process for codesigning an intervention aimed at improving the hospital‐to‐home transition for patients receiving a palliative approach to care and their FCs [11]. By delineating our process, our findings from CDWs to high‐fidelity prototyping sessions can support researchers with future codesigned interventions.

We have centred patients and FCs in the development of the intervention to ensure it meets their needs, while involving HCPs to ensure the intervention fits within their current workflow. Our guidebook intervention will be tested in an implementation pilot study. Pending our results, we hope to integrate the intervention into regular clinical practice in the Champlain Region.

## Author Contributions

**Madeline McCoy:** investigation, formal analysis, visualisation, project administration, writing – original draft, writing – review and editing. **Taylor Shorting:** investigation, formal analysis, visualisation, project administration, writing – review and editing. **Vinay Kumar Mysore:** methodology, investigation, formal analysis, visualisation, project administration, writing – review and editing. **Edward Fitzgibbon:** conceptualisation, investigation, formal analysis, supervision, funding acquisition, visualisation, project administration, writing – review and editing. **Jill Rice:** conceptualisation, investigation, formal analysis, supervision, funding acquisition, visualisation, project administration, writing – review and editing. **Meghan Savigny:** investigation, formal analysis, visualisation, writing – review and editing. **Natalie C. Ernecoff:** methodology, investigation, formal analysis, visualisation, writing – review and editing. **Marianne Weiss:** methodology, investigation, formal analysis, visualisation, writing – review and editing. **Shirley H. Bush:** investigation, formal analysis, visualisation, writing – review and editing. **Daniel Vincent:** investigation, formal analysis, visualisation, writing – review and editing. **Meaghen Hagarty:** investigation, formal analysis, visualisation, writing – review and editing. **Geneviève Lalumière:** investigation, formal analysis, visualisation, writing – review and editing. **Rex Pattison:** investigation, visualisation, writing – review and editing. **Mona Kornberg:** investigation, visualisation, writing – review and editing. **Maya Stern** investigation, visualisation, writing – review and editing. **Kerry Kuluski:** visualisation, writing – review and editing. **Colleen Webber:** visualisation, writing – review and editing. **Adrianna Bruni:** visualisation, writing – review and editing. **Tara Connolly:** investigation, formal analysis, visualisation, writing – review and editing. **Sarina R. Isenberg:** conceptualisation, methodology, investigation, formal analysis, supervision, funding acquisition, visualisation, project administration, writing – review and editing.

## Ethics Statement

This study was approved by the Bruyère Health Research Ethics Board (REB) (study number: M16‐23‐033). The Ottawa Health Science Network Research Ethics Board (OHSN‐REB) determined that this project falls within the context of quality initiative, quality improvement, quality assurance and/or programme evaluation and have exempted this project from requiring ethics approval.

## Conflicts of Interest

The authors declare no conflicts of interest.

## Data Availability

The data that support the findings of this study are available from the corresponding author upon reasonable request.

## Endnotes

1 Family caregivers are defined as people who provide informal support to a patient, but do not need to be directly related to the patient (i.e., they can be the patient's family member, friend, neighbour, etc.).

2 This current study considered transitions from hospital to home, retirement home or group home.

3 Subacute care facilities are intermediate steps between acute care and home, where care is delivered to provide continuity of care for patients.

## References

[1] Canadian Institute for Health Information , 2023, Access to Palliative Care in Canada, https://www.cihi.ca/sites/default/files/document/access-to-palliative-care-in-canada-2023-report-en.pdf.

[2] Canadian Institute for Health Information , 2022, Common Challenges, Shared Priorities: Measuring Access to Home and Community Care and to Mental Health and Substance Use Services in Canada.

[3] B. Gomes , N. Calanzani , M. Gysels , S. Hall , and I. J. Higginson , “Heterogeneity and Changes in Preferences for Dying at Home: A Systematic Review,” BMC Palliative Care 12 (February 15, 2013): 7, 10.1186/1472-684X-12-7. 23414145 PMC3623898

[4] J. D. Kasper , J. L. Wolff , and M. Skehan , “Care Arrangements of Older Adults: What They Prefer, What They Have, and Implications for Quality of Life,” Gerontologist 59, no. 5 (2019): 845–855, 10.1093/geront/gny127. 30476072 PMC6857686

[5] S. F. Jencks , M. V. Williams , and E. A. Coleman , “Rehospitalizations Among Patients in the Medicare Fee‐for‐Service Program,” New England Journal of Medicine 360 (2009): 1418–1428. 19339721 10.1056/NEJMsa0803563

[6] S. Kripalani , C. N. Theobald , B. Anctil , and E. E. Vasilevskis , “Reducing Hospital Readmission Rates: Current Strategies and Future Directions,” Annual Review of Medicine 65 (2014): 471‐85. 10.1146/annurev-med-022613-090415 PMC4104507 24160939

[7] J. Li , R. Young , and M. V. Williams , “Optimizing Transitions of Care to Reduce Rehospitalizations,” Cleveland Clinic Journal of Medicine 81, no. 5 (2014): 312–320, 10.3949/ccjm.81a.13106. 24789590

[8] S. R. Isenberg , T. Killackey , S. Saunders , et al., “‘Going Home [Is] Just a Feel‐Good Idea With No Structure’: A Qualitative Exploration of Patient and Family Caregiver Needs When Transitioning From Hospital to Home in Palliative Care,” Journal of Pain and Symptom Management 62, no. 3 (2021): 9. 10.1016/j.jpainsymman.2021.02.026 33631330

[9] D. A. Knight , D. Thompson , E. Mathie , and A. Dickinson , “‘Seamless Care? Just a List Would Have Helped!’ Older People and Their Carer's Experiences of Support With Medication on Discharge Home From Hospital,” Health Expectations 16, no. 3 (2013): 277–291, 10.1111/j.1369-7625.2011.00714.x. 21838834 PMC5060666

[10] T. Killackey , E. Lovrics , S. Saunders , and S. R. Isenberg , “Palliative Care Transitions From Acute Care to Community‐Based Care: A Qualitative Systematic Review of the Experiences and Perspectives of Health Care Providers,” Palliative Medicine 34, no. 10 (2020): 1316–1331. 32772787 10.1177/0269216320947601

[11] M. McCoy , T. Shorting , V. K. Mysore , et al., “Advancing the Care Experience for Patients Receiving Palliative Care as They Transition From Hospital to Home (ACEPATH): Codesigning an Intervention to Improve Patient and Family Caregiver Experiences,” Health Expectations 27, no. 2 (2024): e14002, 10.1111/hex.14002. 38549352 PMC10979115

[12] D. M. Wilson and S. Birch , “Moving From Place to Place in the Last Year of Life: A Qualitative Study Identifying Care Setting Transition Issues and Solutions in Ontario,” Health & Social Care in the Community 26, no. 2 (2018): 232–239, 10.1111/hsc.12513. 29108131

[13] T. Morey , M. Scott , S. Saunders , et al., “Transitioning From Hospital to Palliative Care at Home: Patient and Caregiver Perceptions of Continuity of Care,” Journal of Pain and Symptom Management 62, no. 2 (2021): 233–241. 33385479 10.1016/j.jpainsymman.2020.12.019

[14] M. Coombs , T. Long‐Sutehall , A. S. Darlington , and A. Richardson , “Doctors' and Nurses' Views and Experience of Transferring Patients From Critical Care Home to Die: A Qualitative Exploratory Study,” Palliative Medicine 29, no. 4 (2015): 354–362, 10.1177/0269216314560208. 25519147 PMC4370931

[15] S. Jones , S. Hamilton , and A. Nicholson , “Rapid Discharge From Hospital in the Last Days of Life: An Evaluation of Key Issues and the Discharge Sister Role,” International Journal of Palliative Nursing 21, no. 12 (2015): 588–595. 26707487 10.12968/ijpn.2015.21.12.588

[16] M. R. Venkatasalu , A. Clarke , and J. Atkinson , “Being a Conduit' Between Hospital and Home: Stakeholders' Views and Perceptions of a Nurse‐Led Palliative Care Discharge Facilitator Service in an Acute Hospital Setting,” Journal of Clinical Nursing 24, no. 11–12 (2015): 1676–1685, 10.1111/jocn.12769. 25736984

[17] S. Saunders , T. Killackey , A. Kurahashi , et al., “Palliative Care Transitions From Acute Care to Community‐Based Care—A Systematic Review,” Journal of Pain and Symptom Management 58, no. 4 (2019): 721–734. 31201875 10.1016/j.jpainsymman.2019.06.005

[18] B. Robert , A. H. Sun , D. Sinden , S. Spruin , and A. T. Hsu , “A Case‐Control Study of the Sub‐Acute Care for Frail Elderly (SAFE) Unit on Hospital Readmission, Emergency Department Visits and Continuity of Post‐Discharge Care,” Journal of the American Medical Directors Association 22, no. 3 (2021): 544–550.e2. 32943339 10.1016/j.jamda.2020.07.020

[19] M. Steen , M. A. J. Manschot , and N. De Koning , “Benefits of Co‐Design in Service Design Projects,” International Journal of Design 5, no. 2 (2011): 53–60.

[20] U.S. Department of Health and Human Services' Agency for Healthcare Research and Quality , *Care Transitions From Hospital to Home: IDEAL Discharge Planning Implementation Handbook* (Agency for Healthcare Research and Quality, 2013), 1–26.

[21] Y. Bombard , G. R. Baker , E. Orlando , et al., “Engaging Patients to Improve Quality of Care: A Systematic Review,” Implementation Science 13, no. 1 (2018): 98, 10.1186/s13012-018-0784-z. 30045735 PMC6060529

[22] S. Donetto , V. Tsianakas , and G. Robert . 2014. Using Experience‐Based Co‐Design (EBCD) to Improve the Quality of Healthcare: Mapping Where We Are Now and Establishing Future Directions.

[23] V. Tsianakas , G. Robert , J. Maben , A. Richardson , C. Dale , and T. Wiseman , “Implementing Patient‐Centred Cancer Care: Using Experience‐Based Co‐Design to Improve Patient Experience in Breast and Lung Cancer Services,” Supportive Care in Cancer 20, no. 11 (2012): 2639–2647, 10.1007/s00520-012-1470-3. 22544223 PMC3461206

[24] S. Silvola , U. Restelli , M. Bonfanti , and D. Croce , “Co‐Design as Enabling Factor for Patient‐Centred Healthcare: A Bibliometric Literature Review,” ClinicoEconomics and Outcomes Research 15 (2023): 333–347, 10.2147/CEOR.S403243. 37220481 PMC10200122

[25] K. F. Giannitrapani , K. Lin , L. A. Hafi , B. Maheta , and S. R. Isenberg , “Codesign Use in Palliative Care Intervention Development: A Systematic Review,” Journal of Pain and Symptom Management 68, no. 4 (2024): e235–e253, 10.1016/j.jpainsymman.2024.06.007. 38909694

[26] B. Hanington and B. Martin , Universal Methods of Design Expanded and Revised: 125 Ways to Research Complex Problems, Develop Innovative Ideas, and Design Effective Solutions, rev. ed. (Rockport Publishers, 2019).

[27] S. Saunders , M. E. Weiss , C. Meaney , et al., “Examining the Course of Transitions From Hospital to Home‐Based Palliative Care: A Mixed Methods Study,” Palliative Medicine 35, no. 8 (2021): 1590–1601. 34472398 10.1177/02692163211023682

[28] M. McCoy , L. Al Hafi , A. Downar , et al., “Facilitating Equitable Subacute‐to‐Home Transitions for Patients Receiving Palliative and/or End‐of‐Life Care: A Literature Review,” Healthy Populations Journal 3, no. 2 (2023): 72–97, 10.15273/hpj.v3i2.11589.

[29] Design Council , Eleven Lessons: Managing Design in Eleven Global Brands. A Study of the Design Process, accessed July 12, 2023, https://www.designcouncil.org.uk/fileadmin/uploads/dc/Documents/ElevenLessons_Design_Council%2520%25282%2529.pdf.

[30] J. Ball . 2019. The Double Diamond: A Universally Accepted Depiction of the Design Process, accessed July 12, 2023, https://www.designcouncil.org.uk/our-resources/archive/articles/double-diamond-universally-accepted-depiction-design-process/.

[31] D. Nessler . 2018. How to Apply a Design Thinking, HCD, UX or Any Creative Process From Scratch—Revised & New Version, accessed July 12, 2023, https://uxdesign.cc/how-to-solve-problems-applying-a-uxdesign-designthinking-hcd-or-any-design-process-from-scratch-v2-aa16e2dd550b.

[32] Miro , 2023, https://miro.com/.

[33] B. M. Bruce Hanington , Universal Methods of Design: 100 Ways to Research Complex Problems, Develop Innovative Ideas, and Design Effective Solutions (Rockport Publishers, 2012).

[34] J. Chipcase , Field Study Handbook (Field Institute, 2017).

[35] B. Hannington and B. Martin , Universal Methods of Design (Rockport Publishers, 2012).

[36] D. Rios , S. Magasi , C. Novak , and M. Harniss , “Conducting Accessible Research: Including People With Disabilities in Public Health, Epidemiological, and Outcomes Studies,” American Journal of Public Health 106, no. 12 (2016): 2137–2144, 10.2105/AJPH.2016.303448. 27736212 PMC5104996

[37] A. S. Williams and S. M. Moore , “Universal Design of Research: Inclusion of Persons With Disabilities in Mainstream Biomedical Studies,” Science Translational Medicine 3, no. 82 (2011): 82cm12, 10.1126/scitranslmed.3002133. PMC3320239 21562227

[38] P. Slattery , A. K. Saeri , and P. Bragge , “Research Co‐Design in Health: A Rapid Overview of Reviews,” Health Research Policy and Systems 18, no. 1 (2020): 17, 10.1186/s12961-020-0528-9. 32046728 PMC7014755

[39] S. Silvola , U. Restelli , M. Bonfanti , and D. Croce , “Co‐Design as Enabling Factor for Patient‐Centred Healthcare: A Bibliometric Literature Review,” ClinicoEconomics and Outcomes Research 15 (2023): 333–347, 10.2147/CEOR.S403243. 37220481 PMC10200122

[40] C. Steele Gray , T. Tang , A. Armas , et al., “Building a Digital Bridge to Support Patient‐Centered Care Transitions From Hospital to Home for Older Adults With Complex Care Needs: Protocol for a Co‐Design, Implementation, and Evaluation Study,” JMIR Research Protocols 9, no. 11 (2020): e20220, 10.2196/20220. 33237037 PMC7725647

[41] K. Okrainec , A. Chaput , V. E. Rac , et al., “Raising the Bar for Patient Experience During Care Transitions in Canada: A Repeated Cross‐Sectional Survey Evaluating a Patient‐Oriented Discharge Summary at Ontario Hospitals,” PLoS One 17, no. 10 (2022): e0268418, 10.1371/journal.pone.0268418. 36194600 PMC9531793

[42] “University Health Network OpenLab Initiative,” About PODS, accessed May 16, 2025, https://pods-toolkit.uhnopenlab.ca/about/.

[43] S. Li , R. Chen , L. Zhang , et al., “Relationships Between Quality of Discharge Teaching, Readiness for Hospital Discharge, Self‐Efficacy and Self‐Management in Patients With First‐Episode Stroke: A Cross‐Sectional Study,” Journal of Clinical Nursing (2024): 1–10, 10.1111/jocn.17481. 39381883

[44] J. Ocloo and R. Matthews , “From Tokenism to Empowerment: Progressing Patient and Public Involvement in Healthcare Improvement,” BMJ Quality & Safety 25, no. 8 (2016): 626–632, 10.1136/bmjqs-2015-004839. PMC4975844 26993640

[45] Y. Y. Tan , Z. Z. Xu , G. S. Pang , et al., “Facilitating Terminal Discharge: Fulfilling the Hospitalised Patient's Wish for Home Death in the Final Hours,” International Journal of Palliative Nursing 22, no. 11 (2016): 541–548, 10.12968/ijpn.2016.22.11.541. 27885905

[46] S. G. R. Barbosa , A. Gavioli , J. R. M. Cicchetto , R. C. N. Sanches , and C. A. T. Radovanovic , “Nursing Checklist of Home Care Guidelines for Informal Caregivers in the Hospital Discharge Transition,” Aquichan 24, no. 1 (2024): 1–18, 10.5294/39111.2024.24.1.3.

[47] E. Sanders , “From User‐Centered to Participatory Design Approaches,” in Design and the Social Sciences: Making Connections, ed. J. Frascara (Taylor & Francis Inc, 2002).

[48] E. B. N. Sanders and P. J. Stappers , “Co‐Creation and the New Landscapes of Design,” CoDesign 4, no. 1 (2008): 5–18, 10.1080/15710880701875068.

[49] Registered Nurses' Association of Ontario , Toolkit: Implementation of Best Practice Guidelines, https://rnao.ca/sites/rnao-ca/files/RNAO_ToolKit_2012_rev4_FA.pdf.

[50] D. Clarke , F. Jones , R. Harris , and G. Robert , “What Outcomes Are Associated With Developing and Implementing Co‐Produced Interventions in Acute Healthcare Settings? A Rapid Evidence Synthesis,” BMJ Open 7, no. 7 (2017): e014650, 10.1136/bmjopen-2016-014650. PMC5734495 28701409

[51] E. Borgstrom and S. Barclay , “Experience‐Based Design, Co‐Design and Experience‐Based Co‐Design in Palliative and End‐of‐Life Care,” BMJ Supportive & Palliative Care 9, no. 1 (2019): 60–66, 10.1136/bmjspcare-2016-001117. 28209568

[52] É. Ní Shé and R. Harrison , “Mitigating Unintended Consequences of Co‐Design in Health Care,” Health Expectations 24, no. 5 (2021): 1551–1556, 10.1111/hex.13308. 34339528 PMC8483209

[53] J. S. Kutner , D. S. Main , J. M. Westfall , and W. Pace , “The Practice‐Based Research Network as a Model for End‐of‐Life Care Research: Challenges and Opportunities,” Cancer Control 12, no. 3 (July 1, 2005): 186–195, 10.1177/107327480501200309. 16062166

[54] N. A. Hagen , P. D. Biondo , P. M. Brasher , and C. R. Stiles , “Formal Feasibility Studies in Palliative Care: Why They Are Important and How to Conduct Them,” Journal of Pain and Symptom Management 42, no. 2 (2011): 278–289, 10.1016/j.jpainsymman.2010.11.015. 21444184

